# Mismatch in microbial food webs: predators but not prey perform better in their local biotic and abiotic conditions

**DOI:** 10.1002/ece3.2236

**Published:** 2016-06-21

**Authors:** Elodie C. Parain, Dominique Gravel, Rudolf P. Rohr, Louis‐Félix Bersier, Sarah M. Gray

**Affiliations:** ^1^ Department of Biology – Ecology and Evolution University of Fribourg Chemin du Musée 10 1700 Fribourg Switzerland; ^2^ Department of Ecology and Evolutionary Ecology Yale University 165 Prospect Street New Haven Connecticut 06520; ^3^ Département de Biologie, Chimie et Géographie Université duQuébec à Rimouski 300 Allée des Ursulines Rimouski Quebec G5L 3A1 Canada; ^4^ Département de Biologie – Ecologie Terrestre Université de Sherbrooke 2500, boulevard de l'Université Sherbrooke Quebec J1K 2R1 Canada

**Keywords:** Consumer‐resource, predator–prey, reciprocal‐transplant experiment, *Sarracenia purpurea*, temperature

## Abstract

Understanding how trophic levels respond to changes in abiotic and biotic conditions is key for predicting how food webs will react to environmental perturbations. Different trophic levels may respond disproportionately to change, with lower levels more likely to react faster, as they typically consist of smaller‐bodied species with higher reproductive rates. This response could cause a mismatch between trophic levels, in which predators and prey will respond differently to changing abiotic or biotic conditions. This mismatch between trophic levels could result in altered top‐down and bottom‐up control and changes in interaction strength. To determine the possibility of a mismatch, we conducted a reciprocal‐transplant experiment involving *Sarracenia purpurea* food webs consisting of bacterial communities as prey and a subset of six morphologically similar protozoans as predators. We used a factorial design with four temperatures, four bacteria and protozoan biogeographic origins, replicated four times. This design allowed us to determine how predator and prey dynamics were altered by abiotic (temperature) conditions and biotic (predators paired with prey from either their local or non‐local biogeographic origin) conditions. We found that prey reached higher densities in warmer temperature regardless of their temperature of origin. Conversely, predators achieved higher densities in the temperature condition and with the prey from their origin. These results confirm that predators perform better in abiotic and biotic conditions of their origin while their prey do not. This mismatch between trophic levels may be especially significant under climate change, potentially disrupting ecosystem functioning by disproportionately affecting top‐down and bottom‐up control.

## Introduction

Climate change is having a strong effect on ecosystems worldwide (e.g., Hughes 2000; Hoegh‐Guldberg and Bruno 2010). Global temperature is expected to increase by 4°C above current average temperatures over the next decades (IPCC 2014), which is predicted to affect organisms according to their level of environmental tolerance (e.g., Pörtner 2001; Deutsch et al. 2008; Somero 2010). Stenothermal species (i.e., species surviving only in a narrow thermal range) may be unable to cope with the changing local environment and thus will either go extinct or need to disperse to suitable environments (Somero 2010). These climate change‐induced range shifts, extinctions, and adaptations may affect biotic conditions by altering the presence and absence of the species in a given area (e.g., Tylianakis et al. 2008), and thus species composition and food‐web dynamics within habitats (e.g., Graham and Grimm 1990; Moritz et al. 2008).

Trophic levels within a food web may be disproportionately affected by climate change, which could ultimately lead to a mismatch between predator and prey dynamics. This mismatch in species interactions could have a large effect on the persistence of a food web and on trophic regulation (e.g., Hoekman 2010). Because climate change is likely to affect species' physiology and composition, it is necessary to investigate the role that abiotic (e.g., temperature) and biotic (e.g., predator–prey interactions) conditions play in creating this mismatch between trophic levels. However, to date, it is not known which of these two conditions (abiotic and biotic) will have the strongest effect on species interactions and species performance at the food‐web level under climate change. Many studies have shown the effect that changes in abiotic conditions have on single species (e.g., Walther et al. 2002; Perry et al. 2005; Chen et al. 2011), while fewer studies have investigated the effect of changes in biotic conditions (e.g., O'Connor 2009; Harley 2011). The combined effect of both abiotic and biotic conditions has, to our knowledge, never been tested. Furthermore, most of the studies addressing changes in the abiotic and biotic environment have focused on only one or two target species (De Block et al. 2013). Yet, species are embedded in communities composed of a complex network of interactions that will all be affected by environmental changes. Consequently, it is now of primary importance to study the effects of environmental change on a whole community in order to develop reasonable and comprehensive recommendations for ecosystem conservation.

This problem is however complex because of the various parameters that environmental change can affect in a diverse community, and the unpredictable consequences due to altered species interactions. A first approach for exploring how abiotic and biotic conditions may affect a whole community is to consider each trophic level as a unit, assuming that the constituting species respond in a similar way to changes. First, with this simplified assumption, the abiotic environment could affect differently the demography of each trophic level (e.g., the intrinsic growth rate and the carrying capacity of prey, the mortality rate of predators), as well as the interaction strength (e.g., through change in attack rate), possibly creating a mismatch if one level is specialized to their local abiotic conditions and the other is not. Second, specialization to biotic conditions could affect the match between the predators and their prey by altering either the rate at which the interaction occurs or the benefit of this interaction to the predator. For these reasons, the outcomes of an experiment are in general difficult to predict without a precise knowledge of how parameters are quantitatively affected by environmental change. In this study, we adopt a parsimonious strategy by considering a trophic level to be specialized when it reaches higher density in the abiotic and/or biotic conditions typically encountered at the location where it was collected (Futuyma and Moreno 1988).

Detection of ecological specialization at the community level also raises methodological issues. As stated above, the global performance of the species composing one trophic level can simply be based on their total density (a measure of the Grinnellian niche). Other possibilities are to use demographic parameters, such as growth rate or mortality, but they are much more challenging to estimate experimentally and to interpret at the community level. Finally, in a predator–prey system, interaction strength (Berlow et al. 2004) is a relevant metric of the Eltonian niche (Devictor et al. 2010), as it is a good descriptor of the efficiency with which energy flows between both compartments.

We investigated the question of the relative effects of abiotic and biotic conditions at the community level using natural aquatic inquiline communities of *Sarracenia purpurea* collected from two sites in Europe and two sites in North America. In each continent, a cold and a warm site were chosen. The *Sarracenia purpurea* communities are mainly composed of bacteria as prey and protists as predators. The species within each trophic levels are highly similar in terms of size and functional role making it possible to treat each trophic level as a unit. Consequently, for tractability in our experiment, we considered the bacteria and the protozoans collectively as two trophospecies (Yodzis and Winemiller 1999), disregarding species‐specific responses within trophic levels. Furthermore, measuring the species‐specific responses would lead to hundreds of variables that would make the interpretation at the community scale intractable.

The *S. purpurea* system has been largely used as an experimental model due to the short generation time of the species, its replicability, and its extensive distribution across North America and Western Europe (Kneitel and Miller 2002, 2003; Miller et al. 2002; Gray et al. 2006; Buckley et al. 2010; Baiser et al. 2012; Krieger and Kourtev 2012). We conducted a reciprocal‐transplant experiment with a factorial design in which the two trophic levels were crossed in local and non‐local biotic and abiotic conditions (four temperatures, four bacteria origins, four protozoan origins) (see Fig. S1, Appendix S1). Additionally, as a control, we grew the bacteria from each origin alone in the four temperatures of origin. First, we studied ecological specialization of bacteria to abiotic condition by measuring their density when grown alone in local versus non‐local temperatures. Second, we investigated the ecological specialization of bacteria and protozoans to abiotic condition by analyzing the change in densities and interaction strength in local versus non‐local temperatures. Third, we investigated the ecological specialization of bacteria and protozoans to biotic conditions by crossing trophic‐level origin and testing how densities and interaction strength were affected. In addition, we evaluated the relative importance of biotic compared to abiotic ecological specialization (i.e., the ability to perform better in local abiotic (temperature) or biotic (presence of local predators and prey) conditions). We estimated the interaction strength using a dynamic index, which is based on the densities of prey (in presence and in absence of predators) and predators, and was developed by Wootton (1997) and Laska and Wootton (1998) (see Methods).

**Figure 1:**
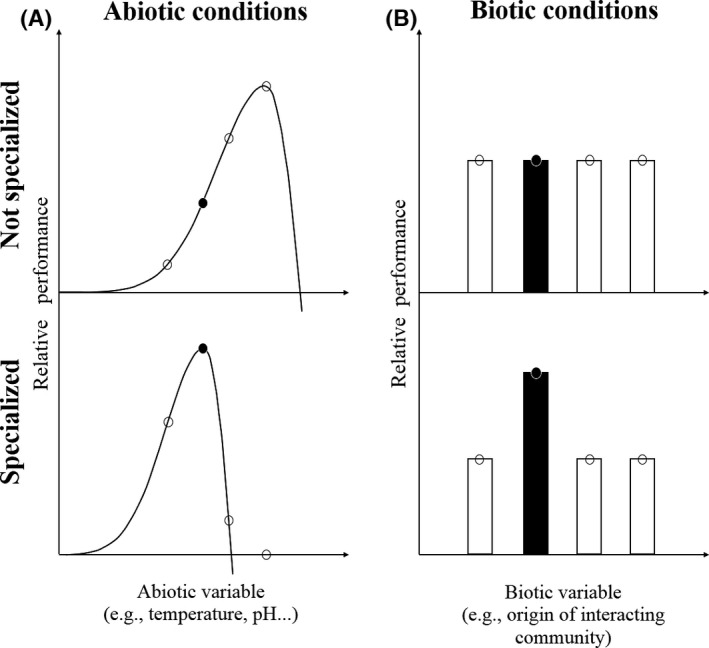
Predicted response in relative performance when species are ecologically nonspecialized versus specialized. The graphic shows the expected relationship between a measure of relative performance (e.g., growth rate) for populations that are ecologically nonspecialized (top row) or specialized (bottom row), to either abiotic (left column) or biotic (right column) conditions. The graphs represent populations tested under different conditions, with the second data point on each graph (in black) being the species in its local condition.

As highlighted above, predictions are in general difficult to formulate because of the many factors that can affect interactions and growth rate within trophic levels. However, based on the general allometry relationship, we predict that there will be a mismatch between the two trophic levels. First, body‐size allometry between predators and prey shows that predators are typically larger bodied (Brose et al. 2006), which is clearly the case in our system; second, Fenchel's allometry demonstrates that larger organisms have a lower maximum rate of increase (Fenchel 1974); third, generation‐time allometry indicates that larger organisms have a longer generation time (Millar and Zammuto 1983). Consequently, larger‐bodied organisms of higher trophic levels should react slower to changing conditions than the smaller‐bodied organisms of lower trophic level. We thus hypothesize that there will be a mismatch between the two trophic levels. The faster‐growing lower trophic level should be better able to track environmental changes, and therefore be less ecologically specialized. Thus, they should perform well in both local and non‐local abiotic and biotic conditions. In contrast, the slower‐growing higher trophic level should be more ecologically specialized and thus perform better in local abiotic and biotic conditions (see Fig. 1). In accordance with our predictions, we will present evidence that the lower trophic levels did not perform better in the local abiotic or biotic conditions of their origin, but that the higher trophic levels performed better and that interaction strength was stronger in local abiotic conditions than in other conditions.

## Methods

### Experimental description

#### Study system

*Sarracenia purpurea* is a pitcher plant species inhabiting nutrient‐poor bogs and thus relies on the capture of insects to fulfill a large portion of its nutrient requirements (Bradshaw and Creelman 1984). The leaves of this plant species, once opened, form a pitcher shape and fill with rainwater. Attracted insects fall in the trap, are decomposed by the microbial community and used as basal nutrients by the plants and bacteria. The bacteria are in the first trophic level of the food chain, and protozoans and rotifers are in the second trophic level (Kneitel and Miller 2002; Karagatzides et al. 2009). A top‐predator mosquito larvae (*Wyeomyia smithii)* prey on all the lower trophic levels (Addicott 1974). In its native range of North America, *S. purpurea* is located from northern Florida, along the eastern United States, and throughout Canada (Schnell 2002).

The plant was introduced approximately 100 years ago into Switzerland (Correvon 1947). This introduction occurred by seed, and the protozoans and top‐predator mosquito species were consequently not dispersed along with the plant. However, bacterivore protozoans of similar phenotype, size, and of the same genus (e.g., *Colpoda*,* Colpidium, Bodo*) as those in North America have established within the leaves of the introduced *S. purpurea* (Fragnière 2012; Zander et al. 2015). These characteristics enabled us to use functionally similar Swiss and North American protozoan communities in our experiment.

#### Experimental design

The experiment consisted of a factorial design including four origins of bacterial and protozoan communities (Florida, Québec and the two Swiss sites: Champ Buet and Les Tenasses) and four incubation temperatures. Site selection was determined by the similarity in the average maximum and minimum temperatures for July according to data obtained from worldclim.org (see details in Appendix S1). We therefore had duplicate native and non‐native sites for the warm and cold temperature limits of the plant species. The warm sites were in Sumatra, Florida (FL, minimum and maximum July temperature: 21.6°C, 32.7°C) and Champ Buet in Switzerland (CB, minimum and maximum July temperature: 18.9°C, 31.4°C). The cold sites were in Saint‐Fabien, Québec (QC, minimum and maximum July temperature: 11.5°C, 22.4°C) and Les Tenasses, Switzerland (LT, minimum and maximum July temperature: 9.2°C, 19.3°C). Four incubators were set to reproduce the maximum, minimum, and mean daily July temperatures for each of the four sites, as indicated by worldclim.org data. Temperature linearly increased from 04 h00 to 16 h00 and decreased over the remainder of the 24‐h period.

To test for ecological specialization to abiotic conditions, bacteria and protozoans from the same origin were grown together in the four different temperatures (one local temperature vs. three non‐local temperatures, see Fig. S1). Specialization to biotic conditions was tested by growing species from one trophic level in their local temperature, but in combination with the predator or prey species from all four possible origins. Consequently, each trophic level from the different origins was grown in its own condition and in all other conditions. As a control, bacteria from each site were also grown in the four temperatures, but without predators (protozoans). We therefore had a total of 28 treatments and 16 controls, replicated four times to make 176 communities (see Fig. S1).

#### Isolating protozoans and bacteria

Four protozoan morphospecies (sizes varying from 10 *μ*m [flagellates] to 30 *μ*m [ciliates]) were isolated from the natural communities originating from the four sites, and are morphologically described as: two flagellates, *Bodo* sp. and one species from the class Chrysophyceae, and two ciliates, *Colpidium* sp. and *Colpoda* sp. To isolate the target species from the other species that were present in the water, we used sterilized deionized water to serially dilute *Sarracenia* water until the protozoan morphospecies of interest was found alone. This dilution was conducted several times to insure that the protozoan of interest was completely isolated from other species. These isolated individuals were then transferred into a tube filled with 1 mL of sterilized deionized water and food (made of a Tetramin fish food solution, Tetra Holding, Blacksburg, VA, USA), according to the protocol given in terHorst (2010). The fish food acted as the basal nutrient input, which was used by bacteria that were isolated along with each protozoan morphospecies; these bacteria served as food for the protozoans. Each isolated protozoan population was stored in an incubator set to the temperature of their original site. They remained in these incubators for 3 days to allow the populations to reach a density of at least 100 ciliates and 5000 flagellates per mL. We periodically checked protozoan populations over 1 week to determine whether they were contaminated with other protozoan species. In case of contamination, we isolated the target species one more time.

In order to isolate the bacteria (size < 0.45 *μ*m) from the four sites, we filtered water with four sterilized vacuum filter devices (one per field site) in which the water passed through three Millipore filter sizes (8 *μ*m, 7 *μ*m glass fiber, and a final passage through a 0.45 *μ*m filter). The filter devices were resterilized after the water passed through each filter size. We fed Tetramin fish food to the bacteria present in the filtered water and stored them in incubators with temperatures matching that of their original sites. After 24 h, the filtered water was checked for contamination of protozoans, in which case the whole filtration process was repeated.

#### Experimental setup

Protozoan communities were assembled by pooling together the four isolated morphospecies from the same site. The water collected from the four field sites was filtered in order to remove all the protozoans and other members of the food web except for the bacterial community. In 50‐mL macrocentrifuge tubes, 10 mL of filtered water containing bacteria at a density of 50,000 individuals per mL was added. We then added the protozoan communities according to the treatments (500 individuals for each flagellate and 10 individuals for each ciliate morphospecies in order to start the experiment with similar biomass). A solution of fish food was added in all the tubes as the basal nutrient input for the communities as it has been shown to have quantitatively similar results as insects (e.g., terHorst 2010; terHorst et al. 2010). We measured the four protozoan morphospecies and bacterial density at the start of the experiment and after 5 days of incubation. Conducting the experiment over 5 days represents approximately 15–20 generations of protozoans (Lüftenegger et al. 1985) and 40 generations of bacteria (Gray et al. 2006). According to Kadowaki et al. (2012) and Gray et al. (2015), 2–4 days are needed for bacteria and protozoans to reach their carrying capacities in this system, and they then plateau for 2–3 days before declining. The fast generation time of microbial systems thus allows experiments to be conducted over a short time period, but yield results equivalent to longer‐term experiments with larger species in term of number of generations (Srivastava et al. 2004). Importantly, the duration of the experiment was set to avoid that the whole system would evolve to laboratory conditions, and thus blur the effect of origin (e.g., terHorst 2010).

### Statistical analyses

#### Specialization to abiotic conditions (temperature)

The aim of this analysis was to test for ecological specialization of bacteria and protozoan communities to abiotic conditions (temperature). A population is specialized when its performance (here estimated by its density) is greatest in its temperature of origin, and in turn, a population is nonspecialized when its density tracks changing temperatures (Fig. 1A). To test for specialization, using density as the dependent variable, we compared the performance of models run with incubator temperature (variable “Temp”) versus absolute difference between origin and incubator temperature (variable “ΔTemp”) as explanatory variables, respectively.

We were first interested in knowing whether the bacteria in the *Sarracenia purpurea* system were specialized in the temperature of their origin. To answer this question, we used bacteria when grown alone (controls) and compared their density (log‐transformed) at the end of the experiment in the different incubators. We measured the performance of the bacteria and protozoan by determining their density at the end of the experiment. In the case of nonspecialization, we expected a positive response to the variable Temp (note that there was no indication of a unimodal response to Temp from visual inspection of the data); in the case of specialization, we expected a negative relationship with the variable ΔTemp. We used linear mixed‐effect model with bacterial group as a random factor (using lme from the nlme package, Pinheiro et al. (2014)). Note that all lme models were run with random intercepts.

We then tested for ecological specialization to abiotic conditions of each trophic level using the treatments where the protozoans and bacteria from the same origin were grown in their local temperature and in the three other non‐local temperatures. We analyzed the results with lme, with origin of bacteria and protozoans as random factors. We used log‐transformed bacterial (protozoan) densities as response variables, and Temp and ΔTemp as explanatory variables, respectively.

#### Specialization to biotic conditions (interacting trophic‐level origin)

In the case of specialization to biotic conditions, we expected that the performance of the trophic level of interest to be greatest in its local biotic conditions (paired with its local predator or prey) than when paired with other non‐local biotic combinations (Fig. 1B). We used a qualitative variable describing if protozoans and bacteria came from the same origin (*Local*) or not (*Away*); a significantly higher value of the response variable would indicate specialization. Note that the *Local*/*Away* variable generates unbalanced data; however, a mixed‐effects model can cope with some degree of unbalance, and we did not encounter convergence issues due to this problem (Zuur et al. 2009). Specialization to biotic conditions was tested for bacteria using treatments where the bacteria were grown in their local temperature, but with protozoans from all origins. To test for specialization to biotic conditions for the protozoans, we used treatments where the protozoans were grown in their local temperature but paired with bacteria from all origins. In both cases, we used lme with bacteria and protozoan origin, respectively, as random factors, and *Local*/*Away* as the explanatory variable.

#### Effects of abiotic and biotic conditions on ecological specialization

In case of specialization to both abiotic and biotic conditions, it is useful to quantify their relative effects in order to better understand how they affect species. A difficulty here was that the statistical approaches used were different for the two types of conditions. To circumvent this problem, we created a factor with three levels for bacteria and for protozoans, which described the match between their origin, the origin of the other trophic level, and of the incubator temperature (*bacteria and protozoans of the same origin in their temperature of origin* (intercept); *bacteria in their temperature of origin with different protozoans* (and accordingly for protozoans)*; bacteria and protozoans from the same origin in different temperatures*). We used lme to relate the log‐transformed densities to this new explanatory variable, with the same random factors as above. We used the estimated parameters of these analyses to quantify and compare the effects of biotic and abiotic conditions on ecological specialization.

#### Effect of abiotic and biotic conditions on interaction strength

We investigated the effect of the abiotic and biotic conditions on the per‐capita interaction strength *γ* between predators (protozoans) and prey (bacteria). Interaction strength was quantified using the index described by Wootton (1997) and Laska and Wootton (1998) (see Appendix S1). The sign of this dynamical index determines the direction of the interaction. A negative value (negative effect) occurs when bacteria (prey) have a lower density when in the presence of protozoans (predators) than when they are grown in the absence of predators. Consequently, we assumed that a more negative interaction strength in local abiotic and/or biotic conditions was indicative that the protozoans, but not the bacteria, were ecologically specialized (or at least that specialization of the protozoans is stronger than specialization of bacteria). A larger value for interaction strength would indicate the opposite effect.

We used a Spearman correlation test with 10,000 permutations when analyzing specialization to abiotic conditions in order to compare the response of the dynamics index to Temp and ΔTemp. A significant positive effect of the latter is indicative of specialization of the protozoans (interaction strength is maximal when there is a match between the origin of the bacteria and of the protozoans). Specialization to biotic conditions was analyzed using a nonparametric Wilcoxon test with *Local*/*Away* as explanatory variable.

All analyses were conducted on the data obtained on the last day of the experiment and were made with the software R (version 3.0.2; R Core Team (2015)). Structure of the residuals was checked with QQ‐plots and Shapiro tests for mixed‐effects models.

## Results

### Specialization of bacteria to abiotic conditions when in absence of protozoans

**Figure 2:**
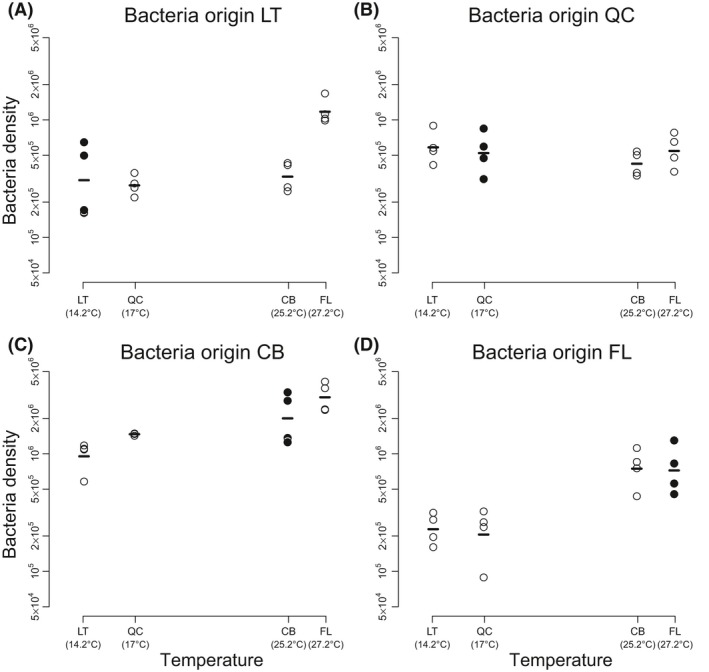
Response of bacteria to temperature when grown without predators. The figure shows the response in density (log‐transformed) of the bacteria from four different origins (panels A–D) to the temperature of those origins. The black dots indicate the case where bacteria were grown in their local temperature. The figure shows that bacteria density responds positively to temperature and not to their local temperature, indicating that bacteria are not specialized. Abbreviations: LT, Les Tenasses; QC, Québec; CB, Champ Buet; FL, Florida.

We found that bacteria density was higher in the warmer temperature treatments, regardless of the origin (Fig. 2). The comparison of the results for the mixed‐effect models that used either ΔTemp or Temp as explanatory variables yielded strong support for the temperature model. However, the variable ΔTemp was negative and statistically significant (ΔTemp: *P*‐value = 0.030; BIC = 154.7; Temp: *P*‐value = 0.009; BIC = 126.5; see Table S1). This result indicated that bacteria were not specialized to their abiotic conditions.

### Specialization of bacteria and protozoans to abiotic conditions

**Figure 3:**
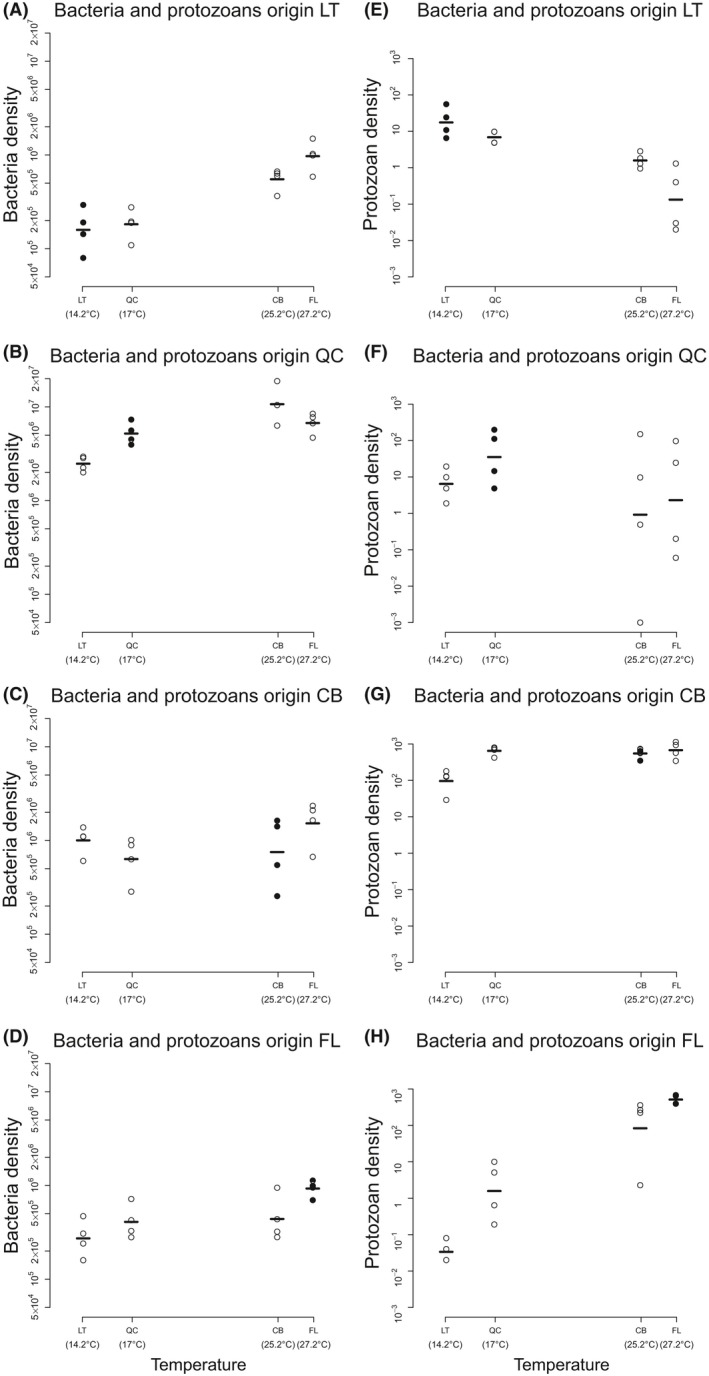
Response of bacteria and protozoans to temperature when grown together. Left column (panels A–D) shows the response of (log‐transformed) densities (individuals/mL) of bacteria for each treatment that contained protozoans and bacteria from the same origin, growing in the four temperatures (*x*‐axis). The origin is given in the panel title; black dots indicate the cases where bacteria and protozoans were grown in their local temperature. Right column (panels E–H): same figure and legend, but for the log‐transformed protozoan densities (individuals/mL). Note that the bacteria and protozoans that are present on the same row in the figure grew in the same tubes during the experiment. This figure shows that bacterial densities increase with temperature but that protozoans have higher densities in their local conditions. Therefore, bacteria are not specialized to abiotic conditions, while protozoans are specialized. Abbreviations as in Figure 2.

We used a subset of the data where protozoans and bacteria were grown together and their origin matched. Based on densities at the end of the experiment, we found discrepant results between the bacteria and the protozoans (Fig. 3). Globally, bacteria densities also increased with Temp in the presence of the protozoans (Temp: *P*‐value < 0.001; BIC = 139.2; ΔTemp: *P*‐value = 0.963; BIC = 167.3; see Table S2), especially for bacteria from extreme temperature sites (LT and FL). In contrast, protozoans had a higher density in their local temperature. We also found a statistically significant effect of temperature on protozoan density. Model selection, however, unambiguously favored the specialization scenario (ΔTemp: *P*‐value < 0.001; BIC = 251.4; Temp: *P*‐value = 0.030; BIC = 284.9; the model including both effects had a BIC = 254.8; see Table S2).

### Specialization of bacteria and protozoans to biotic conditions

**Figure 4:**
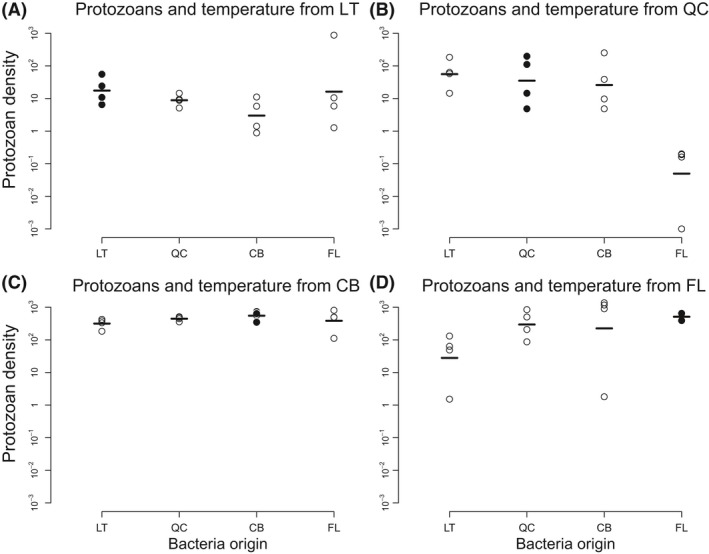
Response of protozoans to biotic conditions. This figure shows the response of (log‐transformed) densities (individuals/mL) of protozoans when grown in their local temperature, in the presence of bacteria from the different origins. The black dots indicate the cases where protozoans were grown in their local temperature with the bacteria from their origin. This figure shows evidence of specialization to biotic conditions for protozoans. Abbreviations as in Figure 2.

We used two subsets of data, one where bacteria were grown in their local temperature but with protozoans from the four origins, and one where protozoans were grown in their local temperature but with bacteria from the four origins. The bacteria did not show evidence of specialization to their biotic condition (Fig. S2, *Local*/*Away*:* P*‐value = 0.465), while the protozoans grew significantly better with bacteria from their origin than with bacteria from the other origins (Fig. 4, *Local*/*Away*:* P*‐value = 0.027; see Table S3).

### Effects of biotic and abiotic conditions on specialization

The protozoans showed a marginally significant effect when in their own temperature but with bacteria from different origins (specialization to biotic conditions; parameter = −0.88, *P*‐value = 0.070), and a highly significant effect when in different temperatures and with their local bacteria (specialization to abiotic conditions; parameter = −2.05, *P*‐value < 0.001, see Table S4), when compared to their densities in local abiotic and biotic conditions. Bacteria densities, in contrast, were never statistically significantly different in local versus non‐local abiotic or biotic conditions (*bacteria and protozoans in their temperature of origin* compared to *bacteria in their temperature of origin with different protozoans*: parameter = −0.03, *P*‐value = 0.93; and to *bacteria and protozoans from the same origin in different temperatures*: parameter = 0.12, *P*‐value = 0.72, see Table S4). Specialization to local abiotic and biotic conditions was thus equally nonimportant for the bacterial trophic level, while for the protozoan trophic level, specialization to abiotic conditions was roughly two times stronger than to biotic conditions.

### Specialization of trophic interaction strength

The variance of the interaction strength parameters was very heterogeneous (see Methods S1 and Fig. S3), precluding the use of mixed‐effect models to analyze specialization of interaction strength to abiotic conditions. Using a Spearman correlation test with permutations provided a straightforward solution for this problem. With a subset of the data where protozoan and bacteria were from the same origin, we found that interaction strength was independent of temperature (Spearman rank correlation between *γ* and Temp, *ρ* = −0.04, *P*‐value = 0.754), but was positively related to ΔTemp, with *ρ* = 0.298, *P*‐value = 0.014 (Fig. S4). In other words, the effect of protozoans on bacteria became weaker when moving away from the local temperature. This result was consistent with protozoans being at an optimum in their local abiotic condition.

To test for specialization to biotic conditions based on interaction strength, we used subsets of data where protozoans were in their local temperature with bacteria from all four origins, and correspondingly for bacteria. Using Wilcoxon tests, we found no evidence of specialization of biotic conditions both for protozoans (W = 410, *P*‐value = 0.495) and bacteria (W = 441, *P*‐value = 0.385) (Figs. S5 and S6). This result suggests that when the protozoans were in their local temperature, they did not consume their bacterial prey at a higher intensity. In addition, the response of the bacteria was independent of predator origin.

## Discussion

Our results provide experimental evidence of a major difference in the response of trophic levels to changes in environmental conditions. The higher trophic level, consisting here of protozoans, performed better in its local abiotic and biotic conditions, while the lower, bacterial trophic level performed consistently better in warmer temperatures. We also found a statistically significant effect of temperature on protozoan density, which can be interpreted as an indirect effect through increased bacteria density, indicating that this system is bottom‐up controlled (Kneitel and Miller 2002). We suggest that the discrepancy between trophic levels' responses is ultimately a consequence of body‐size driven metabolic differences between trophic levels (Gillooly et al. 2001; Brose et al. 2006), and our results from this *Sarracenia* system may thus be of general relevance for community and conservation biology. These results are particularly significant in the context of climate change, as such an observed mismatch in predator–prey dynamics is likely to disrupt ecosystem functioning in many systems, notably by weakening top‐down control. The decrease of predators' performance is likely to release the prey from predation pressure, which will thus be able to increase their densities.

Many studies have focused on the “match/mismatch hypothesis” between predators and prey (Cushing 1974, 1990; Edwards and Richardson 2004; Durant et al. 2007; Schweiger et al. 2008) in an attempt to understand the effect of climate change on interacting species. This effect has been found to occur in a high diversity of taxa in both terrestrial and aquatic systems (e.g., Visser et al. 1998; Durant et al. 2003; Jones and Cresswell 2010; but see Blandenier et al. 2014). However, most of the research was observational and has focused on phenological differences between specific predators and their prey. In the present study, we show that the mismatch between predators is not only a consequence of temporal or spatial divergence, but more generally occurs because the higher trophic level performs less well with non‐local prey communities, or in non‐local temperature conditions. This change is more subtle than temporal or spatial mismatch and will be easily overlooked in the field. However, its effect may be important for community dynamics, with the gradual changes in demographic and interaction parameters possibly leading the system to catastrophic shifts in the longer term (Straile et al. 2001; Stenseth and Mysterud 2002). Ultimately, the system may only persist as one trophic level and thus suffer from a great loss of diversity and complexity.

Our conclusions were based not only on species densities, but also on interaction strength. The estimation of this parameter is however difficult (Wootton and Emmerson 2005), notably because of the high variance associated to this measure when the number of predators is low. Indeed, we encountered problems in our statistical analyses, which could not be solved within the framework of classical mixed‐effects models. Additionally, the interpretation of the results is difficult in the absence of a significant effect, as it may be due to the lack of ecological specialization, as well as to ecological specialization of both protozoans and bacteria. In our case, we were able to detect stronger interaction strength for protozoans in their local abiotic conditions, reinforcing the conclusion reached based on bacteria and protozoan densities. Interestingly, protozoans reached higher densities with their local bacteria, but interaction strength was not different for bacteria from the four origins. This result indicates that protozoans may reach higher densities not because of higher predation rates with their local prey, but because of better efficiency at converting bacterial biomass into protozoan biomass. These results are also consistent with specialization to abiotic conditions being stronger than specialization to the biotic environment. Change in temperature is thus likely to have a stronger effect on community dynamics than change in the composition of the prey trophic level. However, the interpretation of these results needs to be moderated. While temperature is easily controlled, the composition of the bacterial community might have changed during the experiment and affected the detection of ecological specialization to biotic conditions. Finally, note that we cannot attribute our results to particular species dominating at the end of the experiment under the different treatments, as we found a very weak effect of abiotic and biotic conditions on protozoan species composition (See Methods S1 and Table S5 in Appendix S1 for details of canonical correspondence analyses).

To our knowledge, our study is the first experimental attempt to explore, in the context of climate change, the issue of mismatch in a food‐web context in the framework of ecological specialization; no studies have shown that an assemblage of prey can respond differently than an assemblage of predators to changes in temperature and biotic conditions. Other studies have explored the question of “local adaptation” in a trophic context, but by typically examining one prey and one predator species. In a short‐term, reciprocal‐transplant experiment with a single predator species (*Ischnura elegans* larvae) and prey species (*Daphnia magna*) from different origins, De Block et al. (2013) found that prey were thermally locally adapted, unlike in our study. Yet this thermal adaptation disappeared when prey were paired with larvae of the same latitude, suggesting that the predators were also thermally locally adapted. Using a reciprocal‐transplant experiment in a grassland system consisting of several plant species, one orthopteran herbivore and one spider predator, Barton (2011) observed a behavioral response of spiders to increased temperature, which altered their predation on grasshoppers and indirectly affected the plant community. A key difference of these works with our study lies in the time scale relative to generation time: in our experiment, bacteria could achieve 40 generations and protozoans 15 generations. We believe that this feature hints at a basic mechanism for ecological specialization in a food‐web context. Smaller‐bodied organisms with faster generation times should adapt faster to changing conditions than larger‐bodied organisms (Millar and Zammuto 1983). This species‐level adaptation should scale up to ecological specialization at the community level. In food webs, as predators are typically larger than prey (Brose et al. 2006; Naisbit et al. 2011), there will be a delay in the response of higher trophic levels to changing conditions. However, this delay will be observed only if the species do not have time or the capacity to adapt to the environmental changes.

In the present work, we used a simple system amenable to experimentation. However, our approach is not devoid of caveats. For example, we measured protozoan and bacteria densities after 5 days and assumed that they represent equilibrium densities. We chose this time scale as it was found that, in this system, densities stabilize already after approximately 72 h (Kadowaki et al. 2012). Even if this condition was not met, our results would still be valid if the measured densities were proportional to the “real” equilibrium, which is expected in this bottom‐up controlled system when with two trophic levels (Kneitel and Miller 2002). Note that our measure of interaction strength (Laska and Wootton 1998) does not require the equilibrium condition. A second issue is that we consider protozoan and bacteria collectively, as trophospecies. Due to the complexity of the question and of food webs, this approach is a good first step for allowing the study of ecological specialization to abiotic and biotic conditions at the community level. However, this approach lacks information about species composition and identity, which limits our understanding of the mechanisms behind the observed results. Using trophospecies also limits our understanding of the precise interactions between the species of both trophic levels. Such information would be valuable to obtain a detailed picture of the effects of ecological specialization at the community level. Obviously, our microscopic system makes such an undertaking a methodologically daunting task. Moreover, even if such information was available, the number of response variables to consider would make the statistical analyses challenging (not to mention the difficulty to make predictions at the species level). In this respect, our “collective” approach is a sensible strategy to explore the question of ecological specialization at the community level.

We based our arguments on the trophospecies densities at equilibrium and on the trophic interaction strength between our two trophospecies (bacteria and protozoans). Further experiments should be designed to understand how ecological specialization affects demographic parameters that cannot be estimated based on equilibrium densities, for example, intrinsic growth rates, ecological efficiency, and intraspecific competition. By obtaining highly resolved time series data, these parameters could be estimated assuming an underlying dynamical model. A consumer‐resource model with logistic growth of the bacteria (Mouquet et al. 2008) could be a reasonable first step, but it may not be appropriate for bottom‐up controlled systems. A donor‐control model (Arditi and Ginzburg 2012) would be a sensible choice in this case. Even if metabolic theory has been embedded in predator–prey systems (e.g., Brose et al. 2006), and the dependence of interaction strength to temperature has been investigated and modeled (Rall et al. 2010), we still lack a general theory that merges changing abiotic and biotic effects on food‐web dynamics (e.g., Stenseth and Mysterud 2002). Ultimately, the acquisition of highly resolved time series data will inform us on the most appropriate model and will allow the implementation of more effort into modeling in order to understand the mechanisms underlying ecological specialization.

Our results reveal a clear mismatch in ecological specialization between the trophic levels consisting of bacteria and protozoans, which we assume is driven by differences in body size. These results open the door to many interesting research questions: the exploration of the metabolic and behavioral mechanisms underlying ecological specialization of competing predators within a trophic level, the investigation of possible changes in food‐web architecture and their consequences for system dynamics and functioning, the ability and time length needed for which trophospecies can adapt to changing conditions, the scaling‐up of our findings to larger systems and to higher spatial scales in a metacommunity perspective, and the development of dynamical models for the evolution of biotic and abiotic specialization. All these tasks are intriguing and challenging questions of high relevance given current global change and are key to protect natural communities and to prevent the disappearance of species.

## Conflict of Interest

None declared.

## Supporting information


**Appendix S1**
**Methods S1** Additional information on methodological procedures.
**Table S1** Specialization to abiotic conditions for bacteria grown alone.
**Table S2** Specialization to abiotic conditions for bacteria and protozoans.
**Table S3** Specialization to biotic conditions for bacteria and protozoans.
**Table S4** Relative importance of specialization to biotic and abiotic conditions for protozoans.
**Table S5** Results of canonical correspondence analysis.
**Figure S1** Schematic of the factorial experimental design.
**Figure S2** Response of bacteria to biotic conditions.
**Figure S3** Response of interaction strength to abiotic conditions.
**Figure S4** Ecological specialization of interaction strength in abiotic conditions.
**Figure S5** Response of interaction strength to biotic conditions for bacteria.
**Figure S6** Response of interaction strength to biotic conditions for protozoans.
