# Supplementary material for: Mismatch in microbial food webs: predators but not prey perform better in their local biotic and abiotic conditions
Source: Ecol Evol. 2016 Jun 21;6(14):4885–97. doi: 10.1002/ece3.2236 (PMC4979714; doi:10.1002/ece3.2236)
Supplement: Supplementary file 1 — Appendix S1 Methods S1 Additional information on methodological procedures. Table S1 Specialization to abiotic conditions for bacteria grown alone. Table S2 Specialization to abiotic conditions for bacteria and protozoans. Table S3 Specialization to biotic conditions for bacteria and protozoans. Table S4 Relative importance of specialization to biotic and abiotic conditions for protozoans. Table S5 Results of canonical correspondence analysis. Figure S1 Schematic of the factorial experimental design. Figure S2 Response of bacteria to biotic conditions. Figure S3 Response of interaction strength to abiotic conditions. Figure S4 Ecological specialization of interaction strength in abiotic conditions. Figure S5 Response of interaction strength to biotic conditions for bacteria. Figure S6 Response of interaction strength to biotic conditions for protozoans. [file ECE3-6-4885-s001.docx]

**Mismatch in microbial food webs: predators but not prey perform better in their biotic and abiotic conditions**

**Parain Elodie C, Dominique Gravel, Rudolf P. Rohr, Louis-Félix Bersier and Sarah M. Gray**

Methods: Additional information on methodological procedures. 2

Table A1: Specialization to abiotic conditions for bacteria grown alone. 8

Table A2: Specialization to abiotic conditions for bacteria and protozoans. 8

Table A3: Specialization to biotic conditions for bacteria and protozoans. 9

Table A4: Relative importance of specialization to biotic and abiotic conditions for protozoans. 9

Table A5: Results of Canonical Correspondence Analysis. 10

Figure A1: Schematic of the factorial experimental design. 11

Figure A2: Response of bacteria to biotic conditions. 12

Figure S3: Response of interaction strength to abiotic conditions. 13

Figure A4: Ecological specialization of interaction strength in abiotic conditions. 14

Figure A5: Response of interaction strength to biotic conditions for bacteria. 15

Figure A6: Response of interaction strength to biotic conditions for protozoans. 16

**METHODS: Additional information on methodological procedures**

***Sample collection***

The present study was conducted with inquiline communities that were collected from *Sarracenia* leaves at two sites in the native range and two sites in the non-native range of the plant’s distribution. Site selection was determined by the similarity in the average maximum and minimum temperatures for July according to 30 years of data acquired by WorldClim (www.worldclim.org). We therefore had duplicate native and non-native sites for the warm and cold temperature limits of the plant species. The warm sites were Naczi Bog in Sumatra, Florida (FL, native site, 30°16'32"N, 84°50'49"W, minimum and maximum July temperature: 21.6°C, 32.7°C) and Champ Buet in the low elevation of Switzerland (CB, non-native site, 46°36’50’’N, 6°34’50’’E, minimum and maximum July temperature: 18.9°C, 31.4°C). The cold sites were Lac des Joncs in Saint-Fabien, Québec (QC, native site, 48°21'22"N, 68°49'29"W, minimum and maximum July temperature: 11.5°C, 22.4°C) and Les Tenasses in the high elevation of Switzerland (LT, non-native site, 46°29’29’’N, 6°55’16’’E, minimum and maximum July temperature: 9.2°C, 19.3°C) .

Teams in Switzerland, Québec and Florida simultaneously collected water from mixed-aged leaves according to a shared protocol. Each member of the team was trained so that little variation in the collection procedure would occur. At each field site, leaves were randomly selected throughout the site. A sterilized pipette was used to gently mix the aquatic community inside each leaf and deposit it into an autoclaved bottle. The process was continued until 1L of pooled water from all randomly selected leaves was collected. In the native sites, the top predator mosquito larvae were removed from the water immediately after collection. Each of the 4 samples was then distributed in autoclaved bottles with enough oxygen space to allow for 24 hours of travel. The bottles were kept cooled on ice packs to slow community dynamics during transportation. The water collected in Florida and Switzerland was transported overnight to the Université du Québec à Rimouski (UQAR), where the experiment took place. Samples that were collected in Québec remained at 4°C in the laboratory during this time. All permits for collecting and shipping samples were acquired before the start of the experiment.

***Experimental design***

Four incubators were set to reproduce the minimum and maximum daily July temperatures for each of the four sites (Florida: 21.6°C, 32.7°C ; CB: 18.9°C, 31.4°C ; QC: 11.5°C, 22.4°C ; LT: 9.2°C, 19.3°C). Temperature linearly increased from 04h00 to 16h00 and decreased over the remainder of the 24 hour period. The incubators were also set to follow a light:dark cycle of 12 hours, starting at 06h00. Temperature and light conditions inside incubators were checked regularly, allowing us to assume that the experimental error among incubators was negligible compared to the error due to the variability in the response of bacteria and protozoans to the treatments. Inside incubators, tubes were placed in a random block design, with the blocks rotated daily. The experiment lasted for 5 days, or an estimated 15 to 20 generations of protozoans (Lüftenegger et al. 1985) and 40 generations of bacteria (Gray et al. 2006).

***Experimental set-up***

To start with a similar biomass of morphospecies in all replicates, initial population sizes were 500 individuals for each flagellate, and 10 individuals for each ciliate. We used a flow cytometer to measure the bacterial density in the bacteria cultures before the start of the experiment. We then diluted the cultures of the four sites to a standardized concentration of 50'000 individuals of bacteria per mL. We then aliquoted 10 mL of this water into 50 mL macrocentrifuge tubes in which the experiment took place. In each tube, 0.1 mL of water containing the protozoan communities were introduced according to treatment. Note that some contamination by local bacteria was unavoidable at this stage, but was assumed to be negligible due to volume and density differences. A solution of 1 mL of autoclaved Tetramin fish food (concentration of 6 mg of solid fish food in 1 mL of DI water, terHorst (2010)) was added in all the tubes as the basal nutrient input for the communities.

***Monitoring***

We measured protozoan and bacterial density at the start of the experiment and after five days of incubation. After gentle mixing of the community, an aliquot of 100 µL (1% of the total volume; see Palamara et al. (2014)) from each sample was used to count the density of protozoans with a Thoma cell microscope plate. If densities were too low for an accurate Thoma cell microscope plate count, we used an entire microscope slide to count the density of the protozoan in 100 µL. The density of bacteria was measured using a flow cytometer and 100 µL of each sample (Hoekman 2010).

***Statistical analyses: one-tailed tests***

For mixed-effects models using *Temp* or Δ*Temp* as explanatory variables, reported p-values are one-tailed in accordance with the expected sign of the relationship. We chose the best model based on BIC. In practice, when the sign of the relationship was not in the expected direction, we computed the BIC for a model with the intercept only (no explanatory variable), which corresponds to the best model in this situation. It is then necessary to correct its BIC value by addition of the natural logarithm of the number of observations.

***Statistical analyses: dealing with variability in interaction strength***

Interaction strength was quantified using the index described by (Wootton 1997) and Laska and Wootton (1998) with the index calculated as follows:

$$\gamma=\ln\left( \frac{E}{C} \right).\frac{1}{M} ,$$

with *E* the abundance of the bacteria in the presence of protozoans, *C* the abundance of bacteria in the absence of protozoans, and *M* the abundance of the protozoans.

This index is a compound of several measurements (*E*, *C* and *M*), and each has an associated variance. In our case, we have four repetitions of each control density (for each origin), and used their geometric average as *C* in the above equation. Furthermore, the division by *M* strongly influences the variance of *γ*, with low values of *M* generating high variability. In order to try to include this variability in our model we used the varIdent command, and combining it with a varFix variance component assuming it was proportional to (var(*C_i_*)/*M*)^0.5^, with *C_i_* as the four replicates of control density. However, this method was not sufficient to circumvent the high variation issue, therefore we used Spearman correlation tests to analyze our data.

**Impact of abiotic and biotic conditions on protozoan species composition**

We investigated the impact of the abiotic and biotic conditions on the community composition at the end of the experiment with Canonical Correspondence Analysis (CCA). Note that the composition was standardized for all tubes at the start of the experiment. We used the log-transformed densities of the four protozoan morphospecies as response variable, and the binary variables Local/Away for the biotic and the abiotic conditions as explanatory variables. We added protozoan origin as a factor to account for intrinsic site differences. We performed a CCA for each variable to obtain its overall contribution to the total variance of the data, and partial CCA to estimate their exclusive contribution by controlling for both other variables. Analyses were performed with the function cca of the vegan package (Oksanen et al. 2015) in R (R Core Team 2015); the statistical significance of each variable considered globally was evaluated with a permutation test with 10'000 simulations (function anova .cca of the vegan package). The results are given in Table A5.

**REFERENCES**

Gray, S. M., T. E. Miller, N. Mouquet, and T. Daufresne. 2006. Nutrient limitation in detritus-based microcosms in *Sarracenia purpurea*. Hydrobiologia **573**:173-181.

Hoekman, D. 2010. Turning up the heat: temperature influences the relative importance of top-down and bottom-up effects. Ecology **91**:2819-2825.

Laska, M. S., and J. T. Wootton. 1998. Theoretical concepts and empirical approaches to measuring interaction strength. Ecology **79**:461-476.

Lüftenegger, G., W. Foissner, and H. Adam. 1985. r-and K-selection in soil ciliates: a field and experimental approach. Oecologia **66**:574-579.

Oksanen, J., F. G. Blanchet, R. Kindt, P. Legendre, P. R. Minchin, R. O'Hara, G. L. Simpson, P. Solymos, M. H. H. Stevens, and H. Wagner. 2015. Package ‘vegan’. Community ecology package, version:2.2-1.

Palamara, G. M., D. Z. Childs, C. F. Clements, O. L. Petchey, M. Plebani, and M. J. Smith. 2014. Inferring the temperature dependence of population parameters: the effects of experimental design and inference algorithm. Ecology and Evolution **4**:4736-4750.

R Core Team. 2015. R: A language and environment for statistical computing. R Foundation for Statistical Computing, Vienna, Austria.

terHorst, Casey P. 2010. Evolution in response to direct and indirect ecological effects in pitcher plant inquiline communities. The American Naturalist **176**:675-685.

Wootton, J. T. 1997. Estimates and Tests of Per Capita Interaction Strength: Diet, Abundance, and Impact of Intertidally Foraging Birds. Ecological Monographs **67**:45-64.

Table A1 : Specialization to abiotic conditions for bacteria grown alone. Parameter estimates from linear mixed effect models comparing distance to local temperature (Δ*Temp*) and temperature effects on bacteria when grown in the absence of protozoans.

|  | **Random effects** | **Model** | **Fixed effects** | **Estimates** | **SE** | **DF** | **t-value** | **p-value** | **BIC** |
| --- | --- | --- | --- | --- | --- | --- | --- | --- | --- |
| **Bacteria** | Bacteria origin | Δ*Temp* | Intercept | 13.54 | 0.35 | 59 | 38.64 | <0.001 | 154.7 |
|  |  |  | Δ*Temp* | -0.03 | 0.02 | 59 | -1.91 | 0.0030 |  |
|  |  | Temperature | Intercept | 12.04 | 0.64 | 59 | 18.86 | <0.001 | 126.5 |
|  |  |  | Temperature | 0.06 | 0.03 | 59 | 2.41 | 0.009 |  |

Table A2 : Specialization to abiotic conditions for bacteria and protozoans. Parameter estimates from linear mixed effect models comparing distance to local temperature (Δ*Temp*) and temperature effects on bacteria and protozoan densities from a subset of data where protozoan and bacteria origins matched, and the bacteria and protozoan are grown together.

|  | **Random effects** | **Model** | **Fixed effects** | **Estimates** | **SE** | **DF** | **t-value** | **p-value** | **BIC** |
| --- | --- | --- | --- | --- | --- | --- | --- | --- | --- |
| **Bacteria** | Bacteria origin | Δ*Temp* | Intercept | 13.59 | 0.64 | 59 | 21.36 | <0.001 | 167.3 |
|  |  |  | Δ*Temp* | 0.03 | 0.02 | 59 | 1.81 | 0.963 |  |
|  |  | Temperature | Intercept | 12.11 | 0.67 | 59 | 18.08 | <0.001 | 139.2 |
|  |  |  | Temperature | 0.08 | 0.01 | 59 | 6.45 | <0.001 |  |
|  |  | Δ*Temp* + | Intercept | 11.90 | 0.68 | 58 | 17.51 | <0.001 | 143.6 |
|  |  | Temperature | Temperature | 0.08 | 0.01 | 58 | 6.83 | <0.001 |  |
|  |  |  | Δ*Temp* | 0.03 | 0.01 | 58 | 2.60 | 0.99 |  |
| **Protozoans** | Protozoan origin | Δ*Temp* | Intercept | 4.69 | 0.93 | 59 | 5.05 | <0.001 | 251.4 |
|  |  |  | Δ*Temp* | -0.25 | 0.03 | 59 | -7.14 | <0.001 |  |
|  |  | Temperature | Intercept | 1.58 | 1.29 | 59 | 1.22 | 0.11 | 284.9 |
|  |  |  | Temperature | 0.08 | 0.04 | 59 | 1.92 | 0.030 |  |
|  |  | Δ*Temp* + | Intercept | 3.14 | 1.12 | 58 | 2.80 | 0.007 | 254.8 |
|  |  | Temperature | Temperature | 0.07 | 0.03 | 58 | 2.46 | 0.017 |  |
|  |  |  | Δ*Temp* | -0.25 | 0.03 | 58 | -7.37 | <0.001 |  |

Table A3 : Specialization to biotic conditions for bacteria and protozoans. Parameter estimates from linear mixed effect models comparing specialization of bacteria and protozoans to biotic conditions. Using two subsets of data, one where bacteria grew in their own temperature with the different protozoan origins and the second one where protozoans grew in their own temperature with the different bacteria origins. "*Local*" indicates the conditions where bacteria, protozoans and temperature were from the same origins. "*Away*" indicates the cases where the origins of the two trophic levels did not match.

|  | **Random effects** | **Model** | **Fixed effects** | **Estimates** | **SE** | **DF** | **t-value** | **p-value** |
| --- | --- | --- | --- | --- | --- | --- | --- | --- |
| **Bacteria** | Bacteria origin | *Local* vs. *Away* | Intercept (*Away*) | 13.65 | 0.30 | 59 | 45.07 | <0.001 |
|  |  |  | *Local* | 0.03 | 0.33 | 59 | 0.09 | 0.465 |
| **Protozoans** | Protozoan origin | *Local* vs. *Away* | Intercept (*Away*) | 3.90 | 0.90 | 59 | 4.34 | <0.001 |
|  |  |  | *Local* | 0.88 | 0.45 | 59 | 1.97 | 0.027 |

Table A4: Relative importance of specialization to biotic and abiotic conditions for protozoans. Parameter estimates from linear mixed effect models comparing specialization of bacteria and protozoans to biotic and abiotic conditions both expressed as "*Local/Away*" binary variables.

|  | **Random effects** | **Model** | **Fixed effects** | **Estimates** | **SE** | **DF** | **t-value** | **p-value** |
| --- | --- | --- | --- | --- | --- | --- | --- | --- |
| **Bacteria** | Bacteria origin | Biotic and  abiotic conditions | Intercept (specialized to both) | 13.68 | 0.37 | 106 | 36.70 | <0.001 |
|  |  | vs.  Specialized to | Abiotic conditions | -0.03 | 0.34 | 106 | -0.09 | 0.931 |
|  |  | both | Biotic conditions | 0.12 | 0.34 | 106 | 0.36 | 0.722 |
| **Protozoans** | Protozoan origin | Biotic and  abiotic conditions | Intercept (specialized to both) | 4.78 | 0.99 | 106 | 4.84 | <0.001 |
|  |  | vs.  Specialized to | Abiotic conditions | -0.88 | 0.48 | 106 | -1.83 | 0.070 |
|  |  | both | Biotic conditions | -2.05 | 0.48 | 106 | -4.27 | <0.001 |

Table A5 : Results of Canonical Correspondence Analysis (CCA). The overall and exclusive (i.e., controlling for the other variables using partial CCA) contributions of the three explanatory variables are given, with the corresponding statistics and p-values. Percentage contributions are in parenthesis.

|  | **Inertia** | | | |  | **Permutation test** | | |
| --- | --- | --- | --- | --- | --- | --- | --- | --- |
| **Explanatory variable** | **Global** | | **Exclusive** | |  | **Chi2** | **F** | **p-value** |
| **Protozoan origin** | 0.362 | (25.1%) | 0.373 | (25.9 %) |  | 0.362 | 17.10 | <0.001 |
| **Local/Away for biotic conditions** | 0.015 | (1.0 %) | 0.020 | (1.4 %) |  | 0.015 | 1.59 | 0.066 |
| **Local/Away for abiotic conditions** | 0.010 | (0.7%) | 0.021 | (1.5%) |  | 0.010 | 1.09 | 0.160 |
| Total inertia | 1.441 | (100%) |  | |  |  |  |  |

Protozoans from LT, QC, CB or FL

x

Bacteria from LT

Protozoans from LT

x

Bacteria from QC, CB or FL

Bacteria from LT, QC, CB or FL

without Protozoans

Protozoans from LT, QC, CB or FL

x

Bacteria from QC

Protozoans from QC

x

Bacteria from LT, CB or FL

Bacteria from LT, QC, CB or FL

without Protozoans

Protozoans from LT, QC, CB or FL

x

Bacteria from CB

Protozoans from CB

x

Bacteria from LT, QC, or FL

Bacteria from LT, QC, CB or FL

without Protozoans

Protozoans from LT, QC, CB or FL

x

Bacteria from FL

Protozoans from FL

x

Bacteria from LT, QC, or CB

Bacteria from LT, QC, CB or FL

without Protozoans

Les Tenasses (LT)

Québec (QC)

Champ Buet (CB)

Florida (FL)

**Community**

**Temperature**

Figure A1: Schematic of the factorial experimental design. We crossed 4 origins of protozoan communities with 4 origins of bacteria communities (Les Tenassses (LT), Québec (QC), Champ Buet (CB), and Florida (FL) in both cases), and grew each combination in 4 incubators set to the average temperatures of month of July for the 4 sites (LT = 14.2°C, QC = 17°C, CB = 25.2°C, and FL = 27.2°C ). The temperatures varied through time over a cycle of 24 hours (see details in the Methods section).


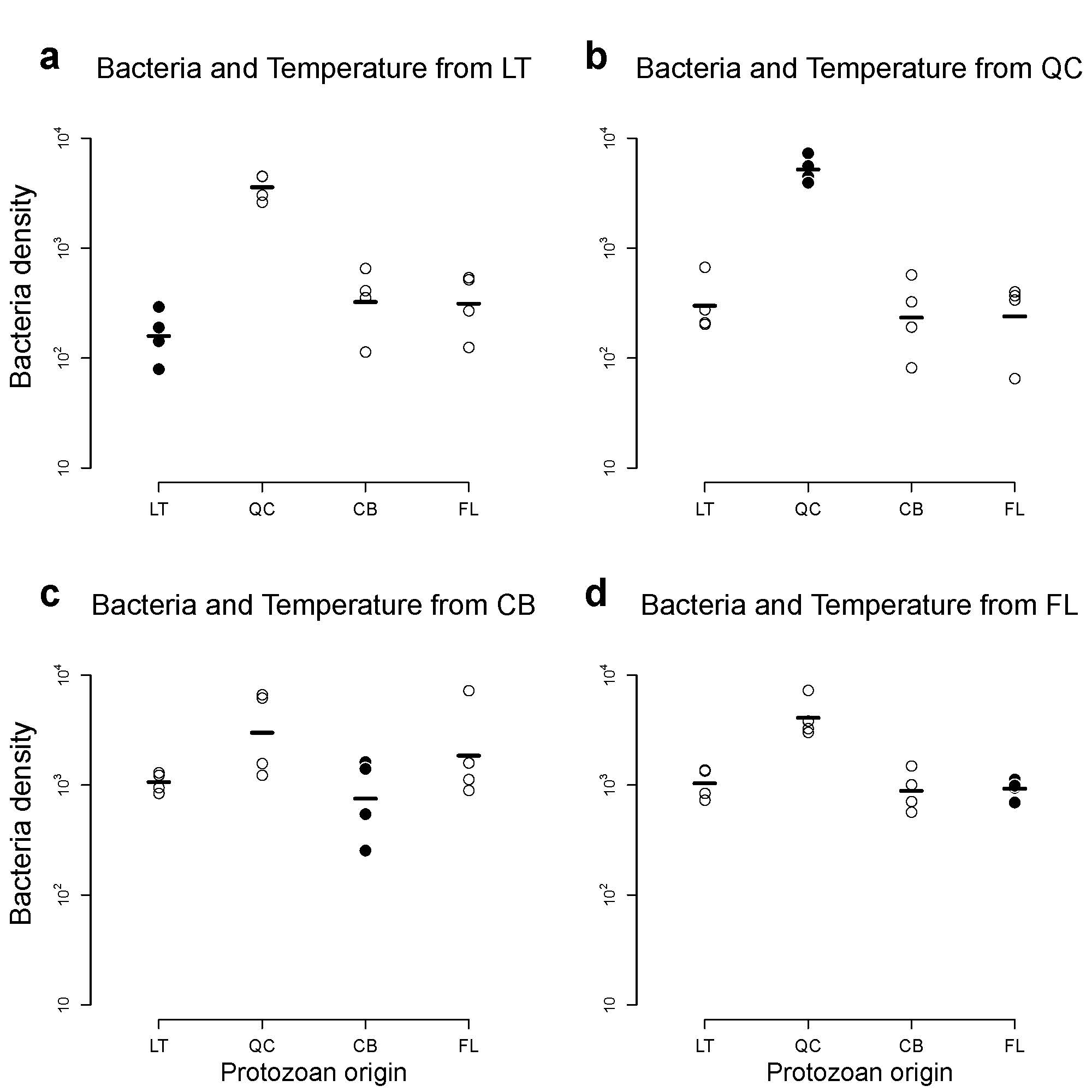


Figure A2: Response of bacteria to biotic conditions. This figure shows the response of (log-transformed) densities (individuals/mL) of bacteria when grown in their local temperature, in the presence of protozoans from the different origins. The black dots indicate the cases where bacteria were grown in their local temperature with the protozoans from their origin. This figure does not show any evidence of specialization to biotic conditions for bacteria. Legend as in Fig. A1.


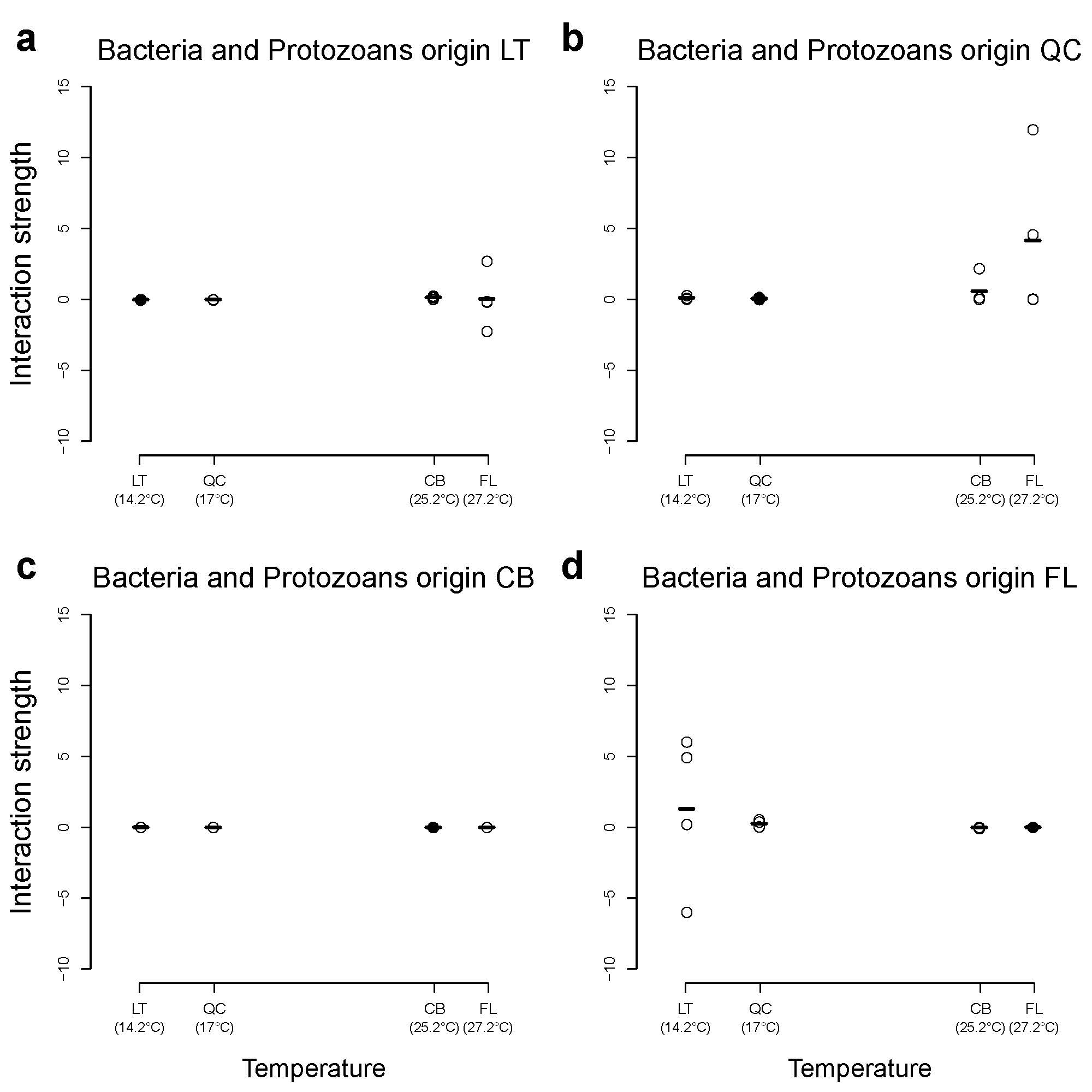


Figure A3: Response of interaction strength to abiotic conditions. This figure shows the response of interaction strength between bacteria and protozoans from the same origins when grown together in the different temperatures. The black dots indicate cases where bacteria and protozoan from the same origin were in their local temperature. This figure illustrates the high variation between each treatment. Note that the estimated values of interaction strength were positive in several cases, indicating that density of bacteria was higher in the presence of protozoans than without. Although we cannot exclude measurement errors, a potential explanation is preferential feeding of protozoan for large bacteria, allowing smaller species to become more abundant. This may lead to a switch towards communities dominated by small species which could have a higher density but a lower biomass than communities with more large bacteria species. Legend as in Fig. A1.


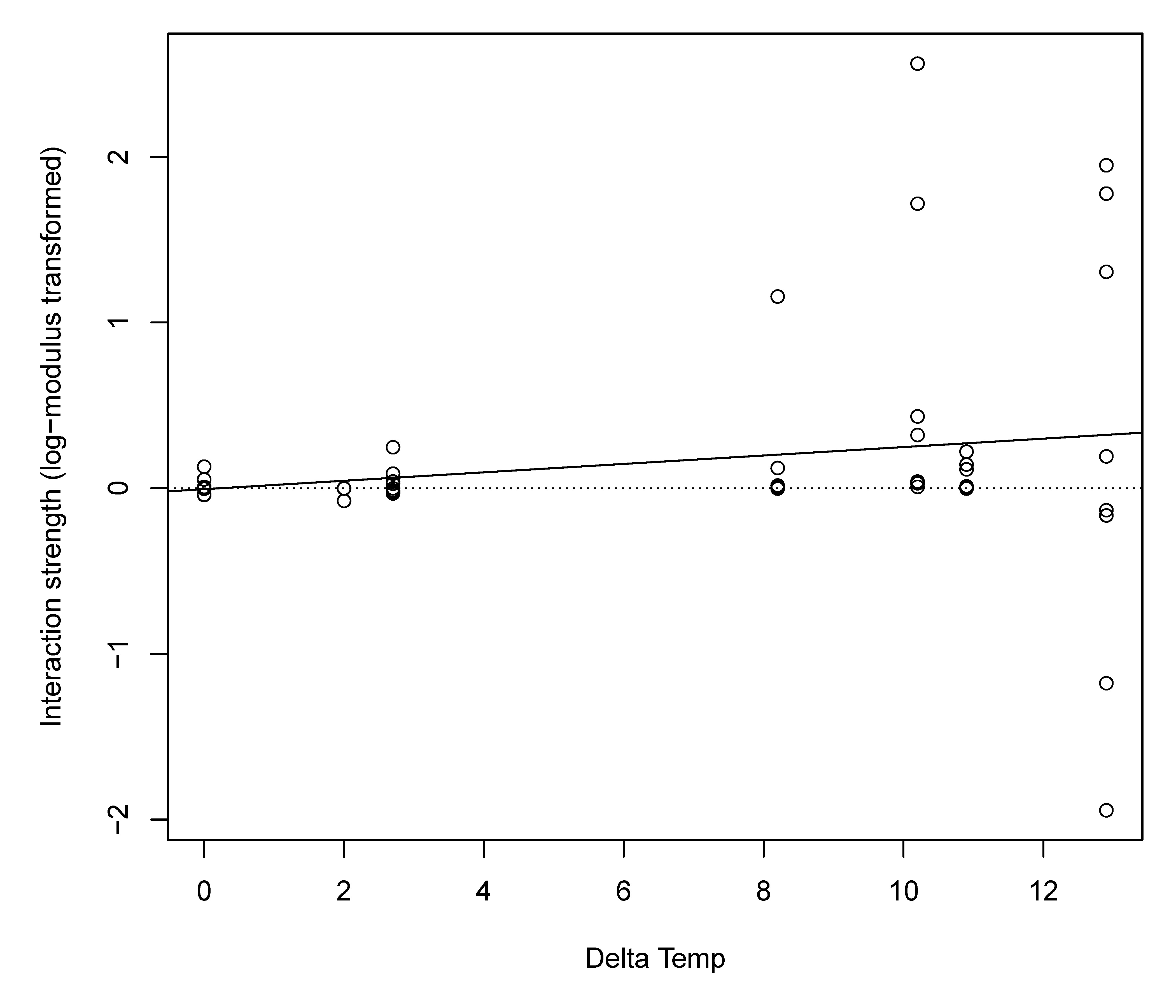


Figure A4: Ecological specialization of interaction strength in abiotic conditions. Using the same data as in Fig. S3, interaction strength (log-modulus transformed) is expressed as a function of Δ*Temp* (Delta Temp). Using Spearman rank correlation, we found that interaction strength was positively related to Δ*Temp*, with *ρ* = 0.298, p-value = 0.014 (p-value from permutation test with 10'000 simulations). The effect of protozoans on bacteria became weaker when moving away from the local temperature, consistent with protozoans being at an optimum in their local abiotic condition. The fitted line is from a linear regression.


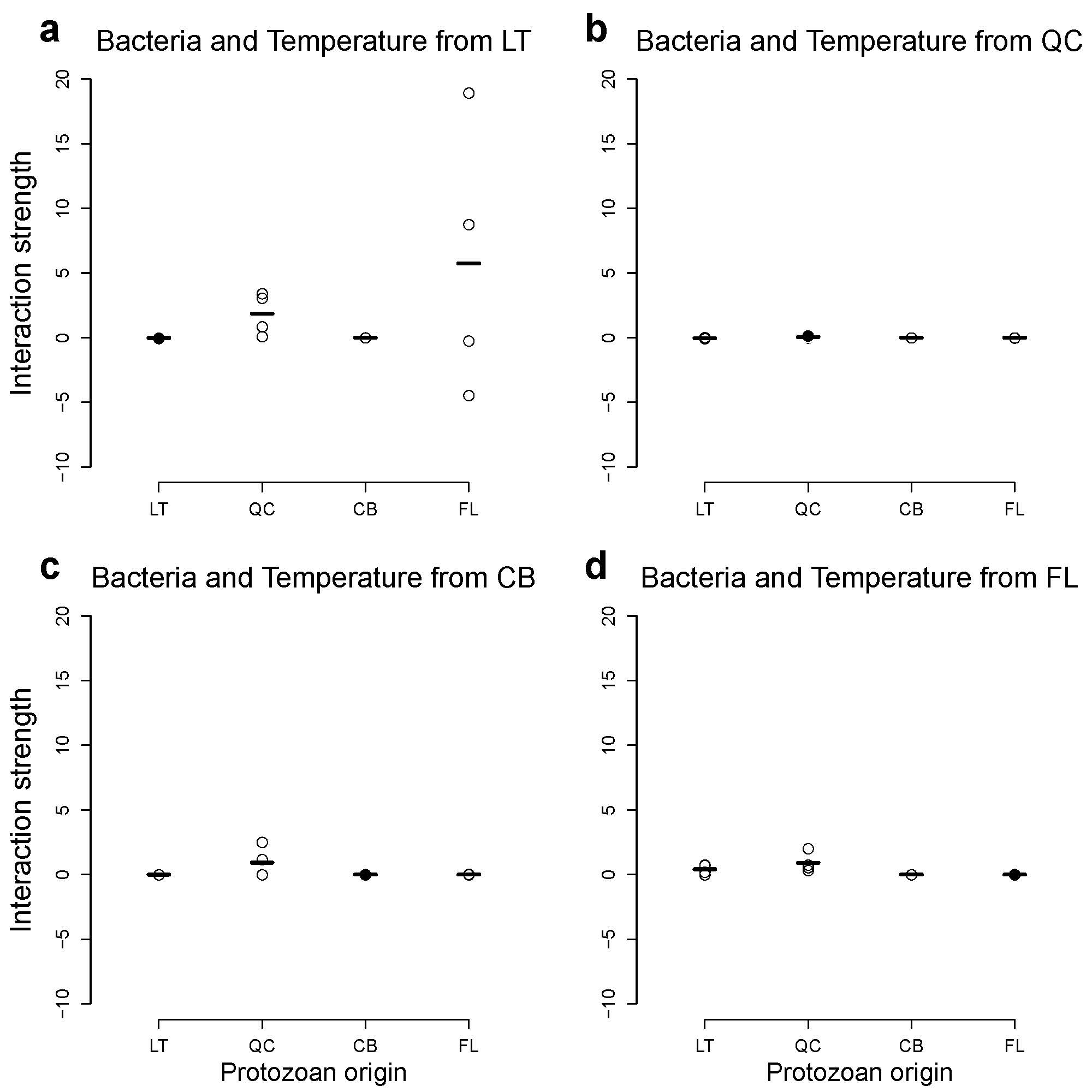


Figure A5: Response of interaction strength to biotic conditions for bacteria. This figure shows the response of interaction strength when bacteria grew in their local temperature, but in the presence of protozoans from the four origins. The black dots indicate the cases where bacteria, protozoan and temperature origins matched. This figure does not show any evidence of specialization of bacteria to biotic conditions. Legend as in Fig. A1.


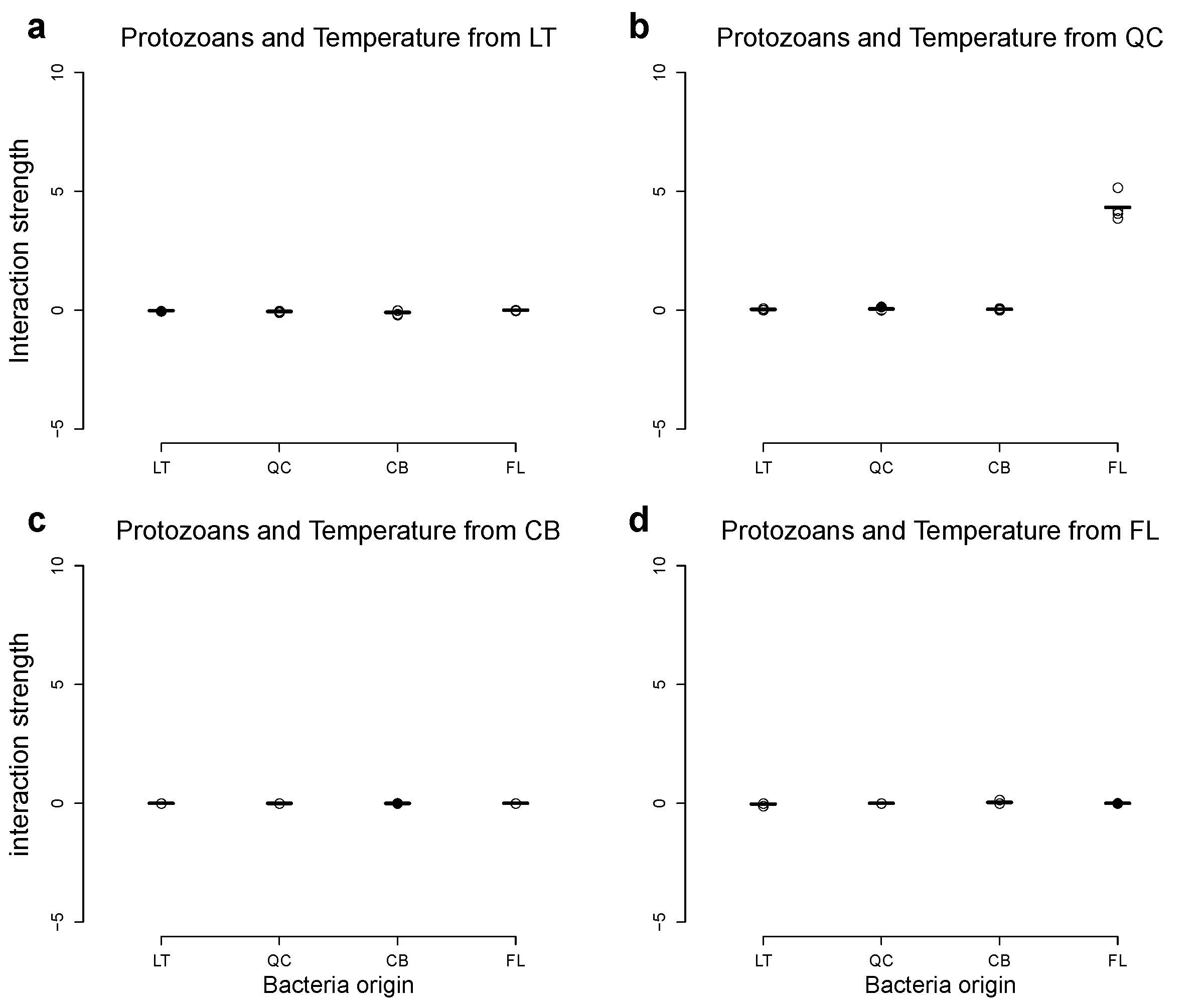


Figure A6: Response of interaction strength to biotic conditions for protozoans. This figure shows the response of interaction strength when protozoans grew in their local temperature, but in the presence of bacteria from the four origins. The black dots indicate the cases where bacteria, protozoans and temperature origins matched. This figure does not show any evidence of specialization of protozoans to biotic conditions. Legend as in Fig. A1.
